# Restorative benefits of multisensory experiences in a classical Chinese garden compared to visual experiences only

**DOI:** 10.3389/fpsyg.2025.1663101

**Published:** 2025-11-28

**Authors:** Yanning Cai, Minkai Sun, Yudie Lu, Yuqin Wang, Seiko Goto

**Affiliations:** 1 School of Environmental Sciences, Nagasaki University, Nagasaki, Japan; 2 Department of Architecture and Urban Planning, Suzhou University of Science and Technology, Suzhou, China

**Keywords:** classical Chinese gardens, mood state, visual perception, multisensory integration, virtual reality, eye movement

## Abstract

**Background:**

Classical Chinese gardens are renowned for their multisensory designs and are widely recognized for their potential to promote emotional well-being. The Humble Administrator’s Garden was utilized in this study to assess how the multisensory integration embedded in its design influences psychological and physiological restoration.

**Methods:**

As a pilot study, a multi-modal quantitative approach compared participants’ responses to the garden under two conditions: a real-world multisensory environment (Condition A) and a visual VR experience using a static 360° image (Condition B). The same group of 28 participants took part in five-minute sessions for each condition. Data included the Profile of Mood States (POMS), heart rate, eye tracking, and a questionnaire. In Condition A, sessions before 8:00 a.m. with few visitors were classified as the “Uncrowded group,” while those after 8:00 a.m., when tour groups arrived, were classified as the “Crowded group,” to assess visitor-related ambient effects on restoration.

**Results:**

Participants in the real-world condition exhibited significantly greater mood modulation, increased attentional engagement, and a reduction in heart rate, particularly in uncrowded settings. In contrast, the VR condition yielded comparatively weaker restorative outcomes, suggesting that both the absence of non-visual inputs and the lack of visual movement limited the replication of the multisensory restorative experience.

**Discussion:**

This pilot study suggests that the restorative effects of classical Chinese gardens derive from the coherence of multisensory inputs, more fully experienced in real settings through visual and non-visual interactions. While multisensory coherence supports psychological benefits, disturbances such as noise and crowding can disrupt this harmony, weakening restorative outcomes. These exploratory findings offer initial guidance for the development of restorative virtual environments and the management of heritage sites, while underscoring the need for larger-scale and more immersive studies.

## 1 Introduction

The restorative effects of natural environments on health are increasingly studied. Many studies, especially those carried out in natural settings, show that exposure to nature can reduce mental fatigue and lower stress (Pretty et al., 2005; Hansmann et al., 2007; Roberts et al., 2019; Lestari and Favurita, 2024). While most focus on visual perception of landscapes, there is growing recognition that multisensory stimulation also significantly enhances nature’s restorative potential (de Wit, 2018; Ratcliffe, 2021; Song and Wu, 2022).

Classical Chinese gardens, especially the private Suzhou gardens of the Ming–Qing period, are renowned for refined, multisensory design grounded in philosophies of human–nature harmony (Cao et al., 2024; Guo et al., 2025; Qi, 2023). Architecture, plants, water features, and rock formations create vibrant, human-centered visual landscapes that support culturally embedded activities (Liang and Feng, 2023). Beyond visual form, these spaces invite reflection and emotional renewal by incorporating curated non-visual cues such as the scent of flowers or the sound of flowing water (Zhang, 2014; Xie et al., 2022; Chen et al., 2021).

The Humble Administrator’s Garden in Suzhou exemplifies the spatial and sensory complexity characteristic of Jiangnan private gardens. Recognized as a masterpiece of classical Chinese landscape design, it integrates architecture, vegetation, water, and pathways into a coherent sensory system that encourages both aesthetic contemplation and emotional restoration (Yu and Fu, 2010; Zhang et al., 2021; Guo et al., 2025). The garden’s layout embodies a balance between visual composition and subtle non-visual cues—such as the spatial rhythm of open and enclosed areas and the interplay of light, shade, and airflow—that together sustain an immersive and tranquil experience (Cen et al., 2023; Chen and Yang, 2023). These features make the Humble Administrator’s Garden an ideal representative site for examining how multisensory coherence contributes to psychological and physiological restoration. As one of the most studied and well-preserved examples of Jiangnan gardens, it provides a culturally and spatially rich environment in which the relationship between sensory integration and restorative outcomes can be empirically tested (Spence, 2020a,b; Zhang et al., 2022).

While classical Chinese gardens engage many senses, it remains unclear how soft, non-visual cues shape where people look and whether those viewing patterns relate to psychological and physiological recovery. To make this connection measurable, we focus on visual perceptual engagement, the process by which attention is directed and maintained on salient garden features. This engagement may serve as a pathway linking multisensory context with restoration (Henderson, 2003; Rayner, 2009). Against this backdrop, the study poses a central question: how are passive and diverse non-visual cues in a classical Chinese garden associated with visual perceptual engagement, and how does that engagement relate to psychological and physiological indices of restoration?

This study empirically investigates sensory engagement in one of China’s most representative classical gardens, the Humble Administrator’s Garden, to clarify how multisensory integration contributes to psychological and physiological restoration. As a pilot study, it establishes a methodological foundation for future, large-scale studies on the restorative potential of classical gardens.

## 2 Literature review

### 2.1 Theoretical foundations of multisensory restoration

Environmental psychology increasingly shows that restoration is not just what we see but what we hear, smell, and feel. Attention Restoration Theory (ART) suggests gentle stimuli trigger involuntary attention, giving focus a break (Kaplan and Kaplan, 1989). Importantly, standard examples are inherently multimodal: the rustling leaves engage hearing and sight, flowing water combines sound with motion. Stress Recovery Theory (SRT) complements this by holding that calm, safe environments reduce arousal and improve mood, seen in heart rate changes (Ulrich et al., 1991).

Within this broader picture, visual attention provides a tractable window into how people engage with complex scenes. The Feature Integration Theory (Treisman and Gelade, 1980) and models of attentional orienting (Posner, 1980) explain how attentional resources are selectively allocated, determining which scene elements are processed in detail and integrated into a percept. Building on these, empirical research shows that gaze metrics—such as fixation duration, fixation frequency, and scan-path distribution—reliably indicate how observers prioritize and extract information (Henderson, 2003; Henderson and Smith, 2009; Rayner, 2009). Crucially, these visual metrics sit alongside non-visual inputs that measurably shape emotion and attention (Baijal and Srinivasan, 2011; Spence, 2020a,b). Multisensory integration theory posits that perception arises from cross-modal interactions, which can enhance processing, engagement, and memory (Angelaki et al., 2009; Calvert et al., 2004; Spence, 2022). Presence theories add a final piece, suggesting that restoration is most likely when cues are sufficiently temporal and spatial to produce a convincing “being there” experience (Slater and Wilbur, 1997; Slater, 2009).

Together, these theories provide a solid conceptual basis for examining how integrated sensory environments, rather than isolated stimuli, support psychological restoration.

### 2.2 Measurement framework

Empirical work on Jiangnan Chinese gardens is limited and methodologically inconsistent. While these gardens integrate vision, sound, scent, and touch into a cohesive experience (Guo et al., 2025), many studies focus on single cues rather than their interactions (Sun and Dong, 2022; Cai and Goto, 2024). This approach understates garden complexity. Methodologically, field studies often employ interviews and questionnaires (Chen and Yang, 2023; Sun et al., 2023; Liu et al., 2024), which capture subjective experiences but lack insight into physiological responses, such as heart rate changes. Lab studies, on the other hand, typically use images or videos for control and physiological measurement but exclude non-visual sensory inputs, reducing ecological validity (Xie et al., 2022; Li et al., 2024; Zhang et al., 2024). Bridging these divides requires designs that assess multisensory cues comprehensively and combine subjective reports with objective physiological data in settings that maintain the authenticity of the garden experience.

Recent methodological advances in environmental psychology now allow more comprehensive and objective assessment of such experiences. Eye tracking quantifies visual attention and attentional stability by measuring where and for how long viewers fixate on landscape elements (Henderson, 2003; Franěk et al., 2018), while heart rate monitoring indexes autonomic responses, with deceleration reflecting parasympathetic activation and physiological relaxation (Ulrich et al., 1991; Goto et al., 2017). When combined with psychological scales, these measures provide a multimodal framework for evaluating restorative effects.

Furthermore, virtual reality (VR) enhances experimental precision by isolating sensory variables while preserving spatial realism. VR enables direct comparisons between real-world multisensory and visual-only experiences (Li and Cao, 2017; Park et al., 2019; Bisso et al., 2020). Although most VR studies employ dynamic, fully immersive simulations, recent evidence shows that even static 360° image-based VR can elicit measurable affective and physiological responses (Brivio et al., 2021). Integrating VR with eye tracking further facilitates controlled observation of visual attention within simulated garden spaces (Shen et al., 2024), enabling reproducible comparisons between multisensory and visual-only conditions in identical settings.

Together, these empirical tools bridge traditional field observation and experimental precision, offering a robust framework for quantifying the psychological and physiological dimensions of multisensory restoration in classical Chinese gardens.

### 2.3 Methodological gap and study objectives

Building on these developments, a key methodological gap remains: most VR-based eye-tracking studies employ dynamic, immersive simulations that include natural motion cues. Such motion—produced by wind, water, or other moving elements—can elicit multisensory expectations (e.g., anticipating sound or airflow), thereby partially reconstructing real-world sensory richness (Adhanom et al., 2023). This blurs the boundary between visual-only and multisensory conditions, leaving a methodological gap that is especially critical in the study of classical Chinese gardens, where sensory integration is central to the experience. Without a clearly defined visual-only baseline, the unique contribution of additional sensory cues to restorative responses cannot be clearly and systematically identified or compared.

To address this gap, this study adopts a multimodal quantitative approach integrating eye tracking, heart rate monitoring, and psychological assessments to compare two conditions at the Humble Administrator’s Garden: (1) a real garden offering immersive, multisensory input, and (2) a VR environment presenting a static 360° image to exclude dynamic motion cues, offering a clear visual baseline, reducing sensory confounds. The study tests the hypothesis that participants will exhibit greater visual engagement, stronger mood improvement, and enhanced physiological relaxation in the real garden compared with the VR condition, due to the combined effects of non-visual sensory cues in promoting restoration.

## 3 Methodology

### 3.1 Site description

**Figure 1:**
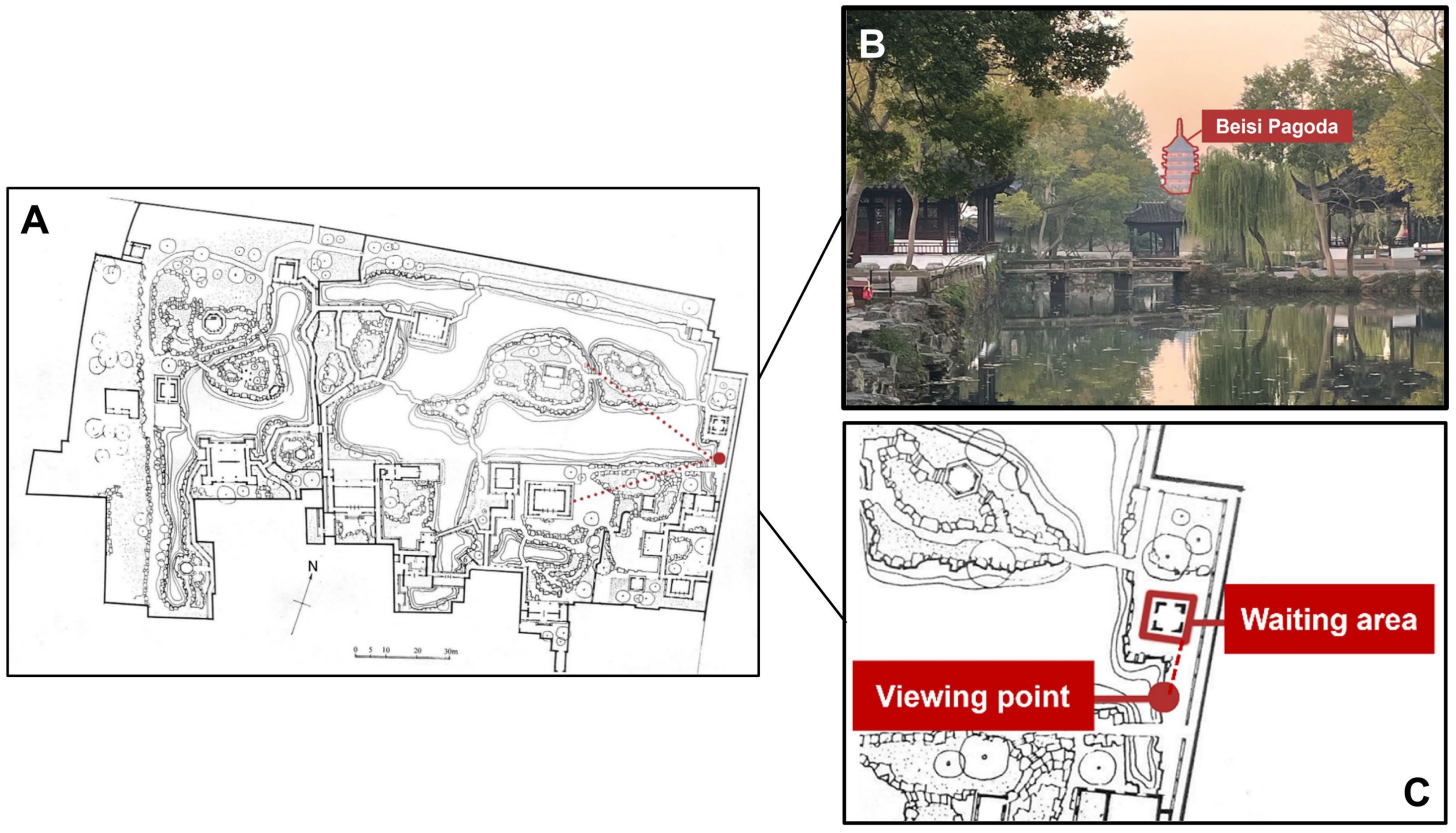
Site. **(A)** The Humble Administrator’s Garden plan and the observation point. **(B)** The view from the observation seat in the Humble Administrator’s Garden. **(C)** Location of observation points and waiting areas.

The Humble Administrator’s Garden was selected as one of the experimental sites because it exemplifies the design principles and spatial organization of classical Chinese gardens (Figure 1A). Recognized as a UNESCO World Heritage Site, it integrates architecture, vegetation, water, and rock compositions into a balanced, human-scaled landscape that embodies the Jiangnan ideal of harmony between humans and nature (Yu and Fu, 2010; Guo et al., 2025).

The chosen observation point for this study is located at one of the most iconic viewing spots in the garden (Figure 1B), featuring a peaceful pond in the foreground, lush trees on both sides, buildings of different heights, and the Beisi Pagoda in the distance, creating a view that incorporates the distant pagoda into the garden scenery (known in landscape design as a ‘borrowed view’) (Sun and Fujii, 2013). The scene offers not only a rich visual hierarchy but also naturally occurring non-visual stimuli that reinforce immersion and reflection. Subtle auditory and olfactory cues—such as the murmur of flowing water, intermittent bird calls, and the faint scent of nearby vegetation—interact with the visual landscape to create a stable multisensory field. Together, these characteristics make this setting ideal for examining how passive, ambient sensory inputs modulate visual perceptual engagement and contribute to restorative experience.

### 3.2 Study design

This pilot study examined the psychological and physiological effects of viewing the same classical Chinese garden—the Humble Administrator’s Garden—under real (Condition A) and virtual (Condition B) settings. The sample size (*n* = 28) was constrained by the one-week experimental period and site accessibility. *A priori* power analysis using G*Power 3.1 confirmed adequacy for detecting a medium effect size (*dz* = 0.6, *α* = 0.05, two-tailed) with a power of 0.80 (critical *t* = 2.07, non-centrality parameter = 2.94).

Twenty-eight undergraduate students (8 males, 20 females; mean age = 23.0 ± 1.5 years) from the Department of Landscape Architecture at Suzhou University of Science and Technology voluntarily participated. Although all had basic training in landscape design, most had limited prior experience with classical Chinese gardens or VR technology. This selection criterion was intended to minimize variability in prior exposure and maintain a consistent cognitive baseline. Participants were randomly assigned to two groups in a counterbalanced repeated-measures design, alternating the order of real and VR experiences across two sessions to eliminate order effects. Individuals with a history of heart disease or best-corrected visual acuity worse than 20/400 were excluded. The Suzhou University of Science and Technology (SUST) Ethics Committee (IRB 190703) approved the study, and all participants provided written informed consent.

Two eye-tracking systems were used: Tobii Pro Glasses 3 (Condition A) and HTC VIVE Pro (Condition B). The Glasses 3 sampled gaze at 100 Hz, and the VIVE Pro Eye at 120 Hz (Appendix A). Heart rate was monitored using IWX/404 fingertip sensors (sampling frequency: 256 Hz) in both settings. Psychological responses were assessed using the Chinese version of the Profile of Mood States (Shacham, 1983) before and after viewing, and a supplemental questionnaire collected participants’ familiarity with classical gardens and VR, along with subjective evaluations of the landscape (Appendix B).

### 3.3 Experimental setup and procedure

The two experimental conditions were conducted in the mornings from October 31 to November 3. Each participant completed the real and VR sessions on separate days, in a counterbalanced order, with an interval of 1–2 days between sessions, depending on their scheduling.

**Figure 2:**
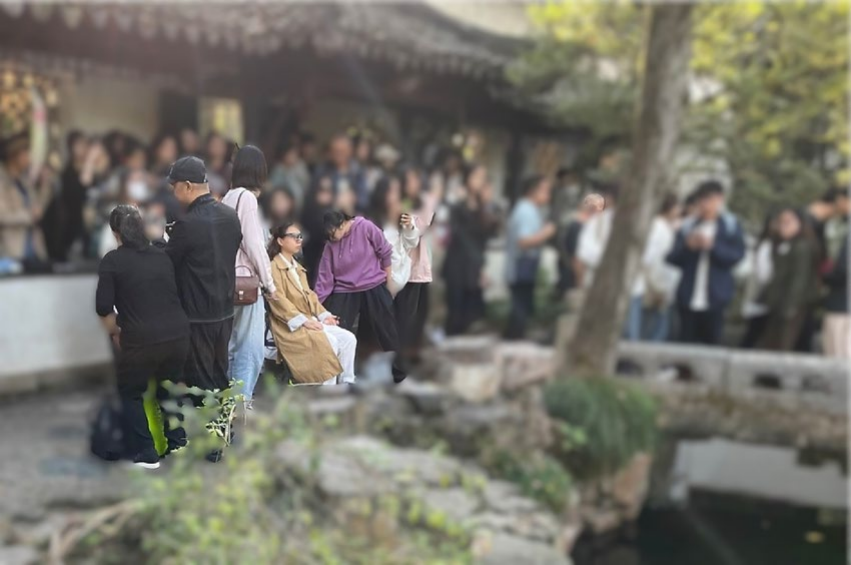
State of the “Condition A” experiment. The experiment was carried out after the garden was opened.

The Condition A experiment was conducted in the Humble Administrator’s Garden under stable weather conditions (20 ± 5 °C; relative humidity ≈ 59%). Participants arrived between 6:30 and 8:30 a.m. and waited in a nearby pavilion before the session began (Figure 1C). To minimize environmental disturbances, the experiment took place during the hour preceding the garden’s public opening (7:30–8:30 a.m.). Prior to 8:00 a.m., the garden was generally quiet, with minimal background noise and few individual visitors. After 8:00 a.m., visitor numbers increased, and large tour groups introduced intermittent acoustic, visual, and olfactory disturbances, including loud conversations, group movements across the viewing area, and occasional scents from food or perfume (Figure 2). Based on these contextual differences, the field data were categorized into uncrowded (before 8:00 a.m.) and crowded (after 8:00 a.m.) periods to compare participants’ responses under varying sensory conditions.

The Condition B experiment took place in a controlled lab at SUST between 8:30 and 10:30 a.m., matching the timing of Condition A to reduce circadian effects. Room conditions were 20 °C with 50% humidity. Participants viewed a single immersive static 360° image of an unoccupied garden, captured at the same viewpoint and time as Condition A, using a head-mounted display with eye-tracking. No sounds were used to focus on visual stimuli, establishing a visual baseline for future research involving more dynamic VR formats.

**Figure 3:**
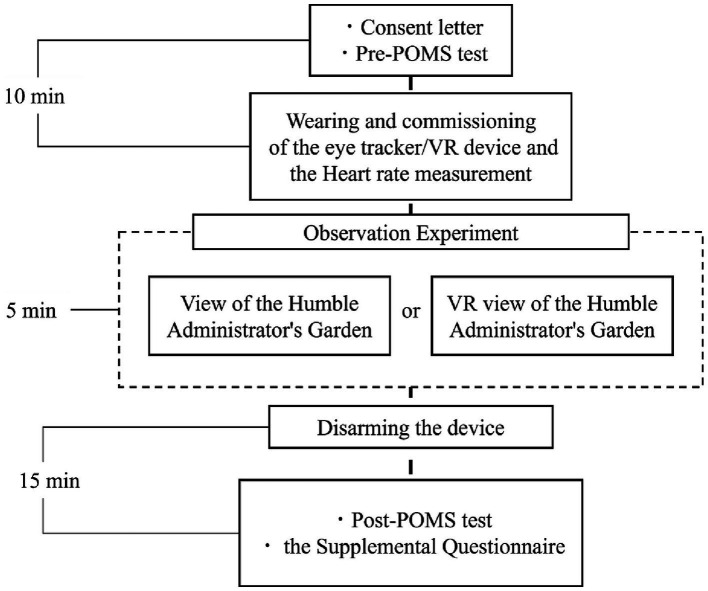
Flow chart of the experiment. The specific flow of each experiment in Conditions A and B.

In both conditions, participants first completed the POMS test in the waiting area. In Condition A, they were then taken to the garden viewpoint and fitted with an eye-tracking device. During the 5-min observation, they remained seated, keeping their gaze within the designated direction to ensure accurate measurement. Condition B followed the same procedure, with participants viewing a VR scene through a head-mounted display with eye tracking. After each session, the equipment was removed, and they returned to the waiting area for a second POMS test and a questionnaire (Figure 3).

### 3.4 Data analysis

**Figure 4:**
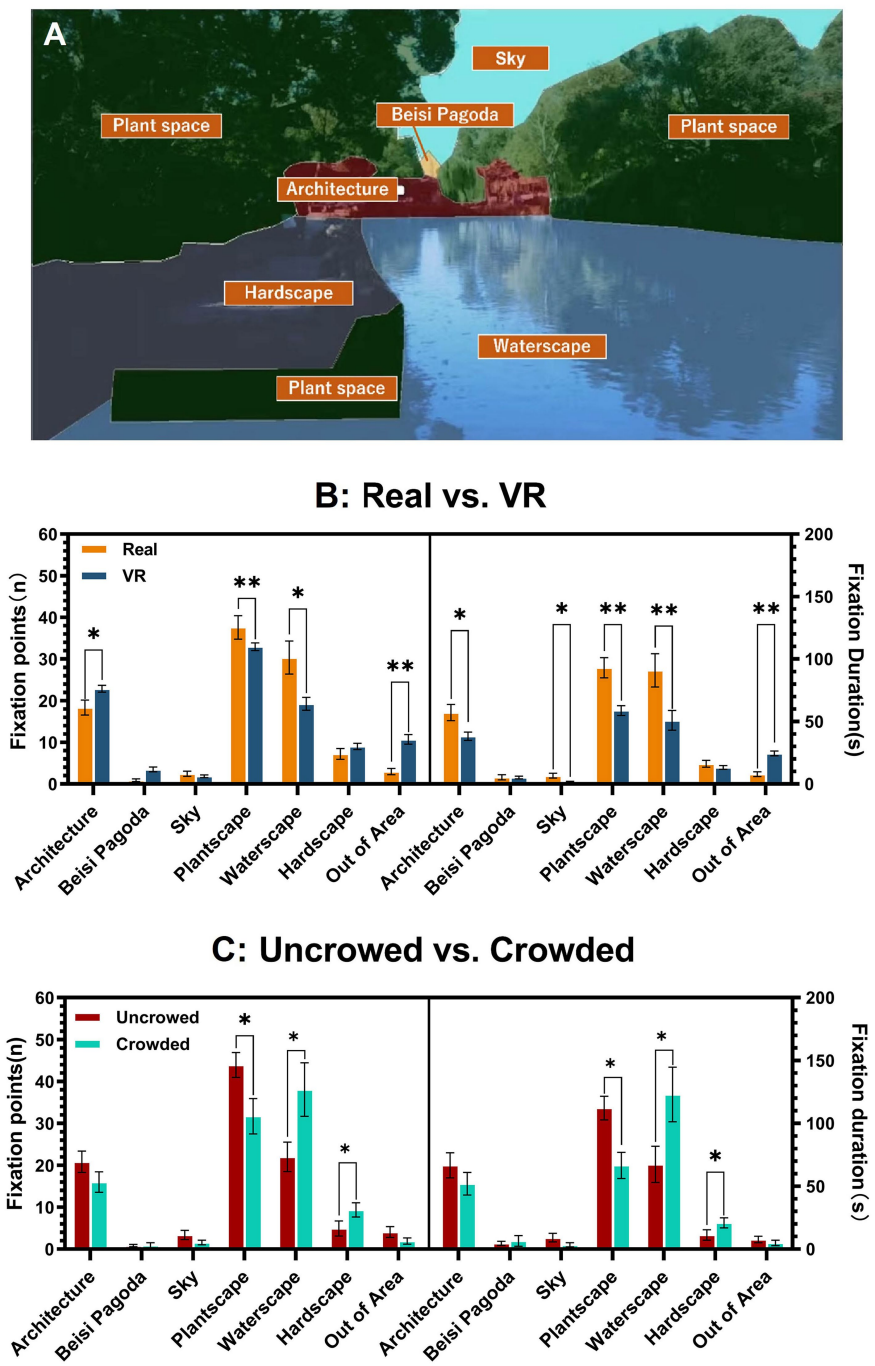
AOIs partitioning and bar graphs. **(A)** The viewing area is divided into six areas of interest, each based on a specific landscape element. **(B)** Comparison of the number of fixations and fixation duration within the AOIs in Conditions A and B (*n* = 26). **(C)** Comparison of the number of fixations and the fixation duration in the AOIs for the Uncrowded and Crowded groups within Condition A (*n* = 13). *p < 0.05, **p < 0.01.

Eye movement data were processed using Tobii Pro Lab software to ensure comparability across conditions, extracting metrics such as gaze range, fixation count, and fixation duration (Marconi et al., 2023). A fixation was defined as ≥200 ms (McConkie and Zola, 1979). The viewing field was divided into six AOIs (Figure 4A), plus an “Out of Area” category for untracked gazes. Heart rate data were processed with Iworx software. The POMS measures six emotional dimensions: five negative (Tension–Anxiety, Depression–Dejection, Anger–Hostility, Fatigue–Inertia, Confusion–Bewilderment) and one positive (Vigor–Activity). To control confounding factors like gender and age, raw scores were converted into standardized T-scores using:


$$T=50+10\times \frac{X-\mu }{\sigma }$$

Where *X* is the participant’s raw subscale total, *μ* is the reference mean, and *σ* is the reference standard deviation from POMS normative data (Terry et al., 1999). Post–pre difference scores were calculated, where negative values indicate beneficial changes for negative mood dimensions and adverse changes for the positive mood dimensions, with the opposite applying to positive values.

After excluding incomplete responses, data from 26 participants were kept for analysis. Additionally, an exploratory analysis was conducted under real-world conditions by categorizing participants into “Uncrowded” (3 males, 10 females) and “Crowded” (4 males, 9 females) groups, based on the division of the timeline (8:00 a.m.).

Two-tailed paired sample t-tests compared eye-movement metrics and POMS data between real and VR conditions. Because of a small sample size (*n* = 13 per group), Wilcoxon rank-sum/signed-rank tests replaced *t*-tests for between- and within-group comparisons to assess visitor presence effects.

Fixation count, fixation duration, and heart rate (HR) were first normalized to each participant’s baseline to enable comparison of relative changes during the 5-min exposure period. Specifically, for each time bin *t*, the value was expressed as a ratio to the participant’s own baseline (the first 30 s), e.g., HR ratio_(t)_ = HR_(t)_/HR _(baseline)_. These ratios were analyzed using two-way repeated-measures ANOVA (time × condition) and two-way mixed-design ANOVA (time × crowding). When ANOVA revealed significant omnibus effects, simple-effects or trend tests were conducted as appropriate. All *post hoc* and simple-effects *p* values were Bonferroni-adjusted within their comparison family (adjusted *p*-values were reported as $p_{\mathrm{adj}}$ ).

Responses to the supplemental questionnaire (Q2–Q4, see Appendix B) used a five-point Likert scale from −2 to +2. For example, Q2 asked, “I like the view of the garden,” with options: strongly agree (+2), agree (+1), neutral (0), disagree (−1), strongly disagree (−2). Fisher’s exact tests examined group differences in responses. An additional analysis was performed to explore the correlations between the questionnaire scores and heart rate decline ratio, which represents the relative change during the 5-min observation: HR_(final)_ – HR_(baseline)_/HR_(baseline)_. HR_(final)_ is the mean in last 30 s.

Statistical analysis used Prism 7.0 (GraphPad). Paired *t*-tests, Wilcoxon tests, ANOVA tests and Pearson correlations were applied; all two-tailed at *α* = 0.05. Effect sizes included *Cohen’s dz*, *Rosenthal’s r*, *ηp*^2^, and *Pearson’s r* with 95% confidence intervals. *Post hoc* power analysis used G*Power 3.1, based on the paired-sample t-test model with two-tailed comparisons.

## 4 Results

### 4.1 Profile of mood states

**Figure 5:**
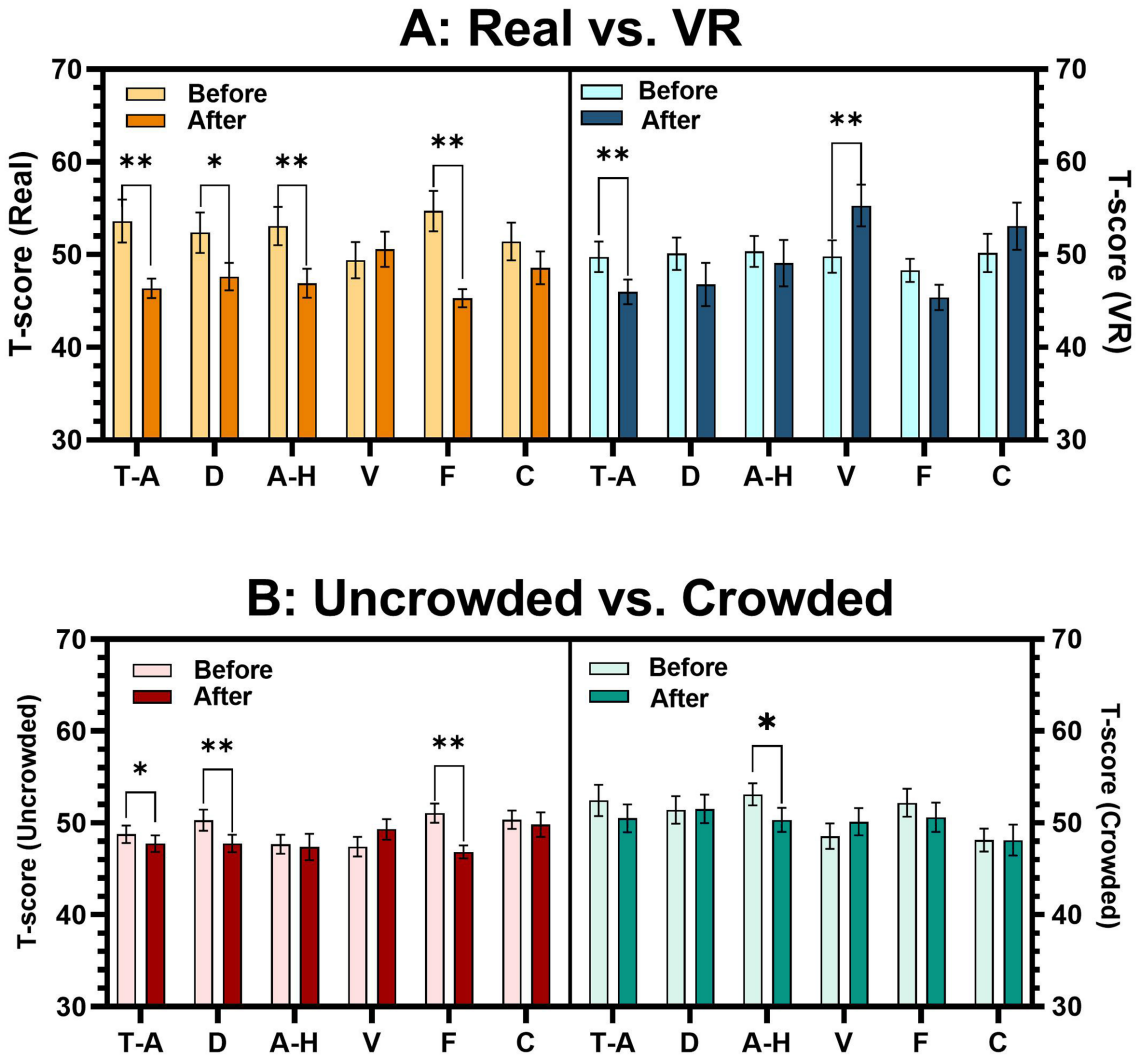
Bar graph of POMS. **(A)** Comparison of the POMS score in Conditions A and B (*n* = 26). **(B)** Comparison of the POMS score for the Uncrowded and Crowded groups within Condition A (*n* = 13). *p < 0.05, **p < 0.01, T-A, tension-anxiety; D, depression-dejection; A-H, anger-hostility; V, vigor-activity; F, fatigue-inertia, and C, confusion-bewilderment.

Table 1A compares mood changes (post–pre) under both conditions using two-tailed paired-sample *t*-tests, showing that real-world garden exposure resulted in greater reductions in Anger and Confusion compared to VR viewing. Figure 5A shows decreases in Tension [*p* = 0.003, *dz =* 0.616, *power* = 0.855, *t(25)* = −3.141], Depression [*p =* 0.043, *dz =* 0.587, *power* = 0.820, *t(25)* = −2.990], Anger [*p* = 0.007, *dz =* 0.589, *power* = 0.823, *t(25)* = −3.00], and Fatigue [*p <* 0.001, *dz =* 0.848, *power* = 0.985, *t(25)* = −4.32]. By contrast, VR exposure produced significant beneficial changes, specifically reductions in Tension [*p* = 0.041, *dz =* 0.435, *power* = 0.563, *t(25)* = −2.218] and Fatigue [*p* = 0.019, *dz =* 0.492, *power* = 0.673, *t(25)* = −2.508]. These results suggest both modalities are beneficial to mood, but the real-world multisensory environment offers significantly greater psychological benefits than visually isolated VR.

**Table 1:** Results of profile of mood states questionnaire (post–pre).

(A) Results of profile of mood states questionnaire in the experiments of Condition A and B, using two-tailed paired-sample *t*-tests								
Mood states	Mean ± SD (Real)	Mean ± SD (VR)	Mean Diff. (Real – VR)	SE Diff. (Real – VR)	*t* (df = 25)	*p*	Effect size (*dz*)	Power
T-A	−5.947 ± 10.63	−4.645 ± 6.63	−1.302	0.852	−1.525	0.14016	0.299	0.311
D	−4.191 ± 13.88	−3.356 ± 10.30	−0.835	1.079	−0.769	0.449	0.151	0.114
A-H	−6.615 ± 9.47	−1.114 ± 8.00	−5.509	2.143	−2.571	0.016*	0.504	0.695
V	0.948 ± 9.61	1.654 ± 7.93	−0.796	0.804	−0.871	0.392	0.171	0.133
F	−8.840 ± 11.27	−8.959 ± 10.06	0.119	0.265	0.452	0.655	0.089	0.072
C	−2.434 ± 7.15	−1.236 ± 6.61	−1.192	0.434	−2.742	0.011*	0.538	0.751

(B) Results of profile of mood states questionnaire for the uncrowded and crowded group within Condition A, using the Wilcoxon rank-sum test								
Mood states	Mean ± SD (Uncrowded)	Mean ± SD (Crowded)	Mean Diff. (Uncrowded – Crowded)	*U*	*Z*	*p*	Effect size (*r*)	Power
T-A	−1.026 ± 9.328	−1.940 ± 9.428	0.914	69.500	0.776	0.439	0.152	0.061
D	−2.530 ± 6.230	−0.121 ± 9.054	−2.409	81.000	0.176	0.859	0.035	0.107
A-H	−0.294 ± 4.024	−2.778 ± 5.932	2.484	26.500	2.967	0.003**	0.582	0.819
V	1.887 ± 9.748	1.565 ± 16.002	0.322	82.000	0.126	0.899	0.025	0.053
F	−4.222 ± 7.010	1.571 ± 7.236	−5.793	46.500	1.943	0.052	0.381	0.344
C	−0.542 ± 11.399	0.004 ± 10.942	−0.546	83.500	0.063	0.947	0.012	0.052

T-A, tension-anxiety; D, depression-dejection; A-H, anger-hostility; V, vigor-activity; F, fatigue-inertia; and C, confusion-bewilderment (*p < 0.05, **p < 0.01).

Further analysis with the Wilcoxon test shows that, although Table 1B suggests greater anger reduction in crowded conditions, this may be due to participants in the crowded group starting with higher initial anger levels, as indicated by baseline values in Figure 5B. The study’s crowded condition involved a high influx of visitors, causing multiple simultaneous disturbances that may have influenced participants’ affective states even before the observation session began. Figure 5B also show that participants in quieter conditions experienced greater reductions in Depression (*p* = 0.002, *r* = 0.857, *power* = 0.782, *Z* = 3.09) and Fatigue (*p* = 0.009, *r* = 0.724, *power* = 0.857, *Z* = 2.610), indicating that high visitor density not only alters baseline emotional states but can also diminish the extent to which garden exposure alleviates negative mood, thereby limiting the overall restorative potential of the environment.

### 4.2 Heart rate

**Figure 6:**
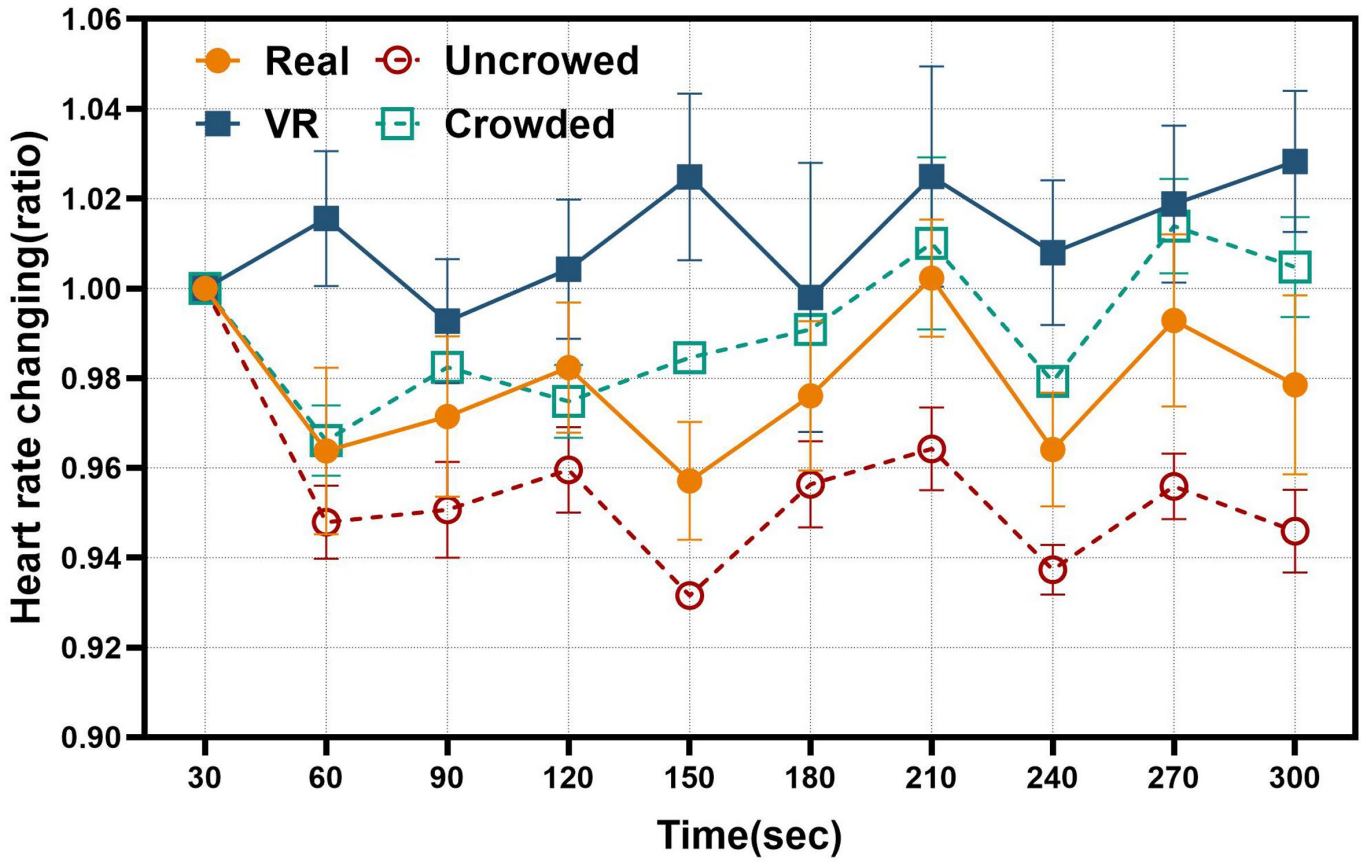
Line graphs of heart rate. Comparison of the heart rate changes every 30 s.

Despite visual differences in heart rate ratio trajectories across conditions (Figure 6), a two-way repeated-measures ANOVA on HR ratios (time × condition; 10 bins of 30 s) detected no main effects of time or condition and no time × condition interaction (all *p*s > 0.05). This indicates that HR did not change reliably over time and that temporal patterns were comparable between the real-world and VR settings.

Within Condition A, we compared the uncrowded and crowded subsets using the two-way mixed-design ANOVA model. A significant main effect of time emerged only in the Uncrowded group [*p* = 0.008, *ηp^2^* = 0.180, *power* = 0.758, *F(9,108)* = 2.64], with Bonferroni-adjusted *post hoc* contrasts showing that HR during the second and last 30-s bins were lower than the initial bin (60s: unadjusted *p* < 0.001; $p_{\mathrm{adj}}$ = 0.003; 300 s: unadjusted *p* = 0.020, $p_{\mathrm{adj}}$ = 0.041). No other pairwise differences survived correction. The Crowded group showed no significant time-related change [*p* = 0.381, *ηp^2^* = 0.083, *power* = 0.416, *F(9,108)* = 1.09].

Taken together, these results suggest a significant but modest decline in heart rate over time only under uncrowded conditions, consistent with a relaxation response; this pattern was not observed in the VR or crowded settings.

### 4.3 Eye movement

**Figure 7:**
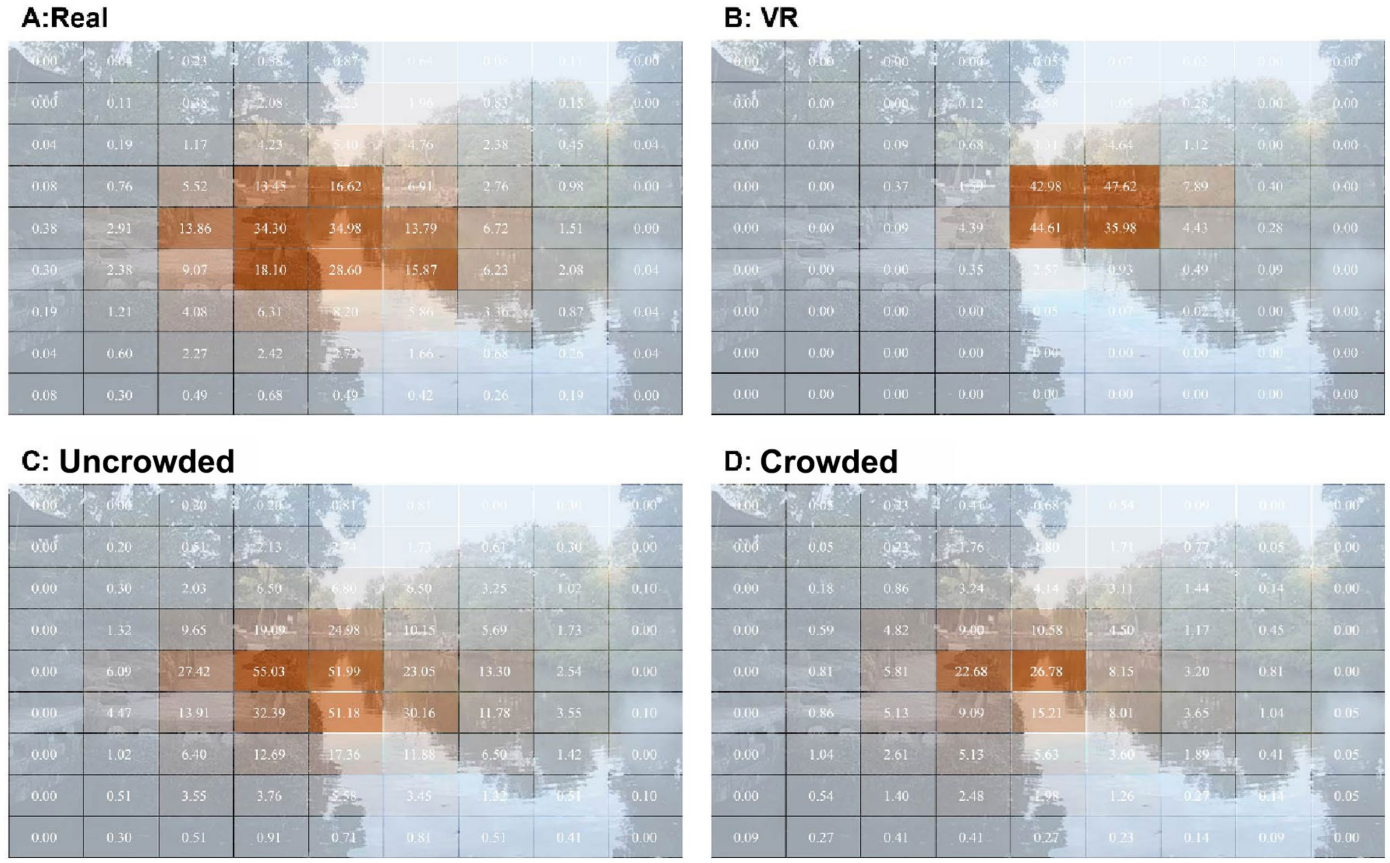
The average fixation duration of all valid samples in a 9 × 9 grid. **(A,B)** The average duration of fixation in each grid region in Condition A and Condition B (*n* = 26). **(C,D)** The average duration of fixation in each grid region of the Uncrowded and Crowded groups within Condition A (*n* = 13).

Gaze behavior differences between settings were analyzed using paired *t*-tests. No significant difference in fixation count was found, but fixation durations were longer in Condition A than in Condition B [Table 2A; *p* < 0.001, *dz =* 1.044, *power* = 0.999, *t(25)* = 5.323]. Both the mean fixation duration and the fixation ratio (the proportion of total viewing time spent fixing on AOIs) were higher in Condition A [Mean duration *p <* 0.001, *dz =* 0.871, *power* = 0.992, *t(25)* = 4.441; Ratio of fixation duration *p* < 0.001, *dz =* 0.800, *power* = 0.979, *t(25)* = 4.079], consistent with heatmap patterns: wider, evenly distributed gaze in Condition A versus central focus in Condition B (Figures 7A,B). In Condition A, warm-colored cells (indicating longer fixations) covered a larger area, showing a wider gaze spread than in Condition B. In Condition B, warm areas were mainly central, revealing a narrower focus. Within Condition A, the Uncrowded group had longer fixation durations (Table 2B; *p* = 0.035, *r* = 0.574, *power* = 0.708, *Z* = 2.116) and a higher fixation ratio (*p* = 0.036, *r* = 0.565, *power* = 0.775, *Z* = 2.093) than the Crowded group, aligning with their broader coverage of warm-colored regions in heatmaps (Figures 7C,D). Participants in Condition A showed longer, more dispersed fixations, especially uncrowded, while Condition B exhibited more central, limited gaze behavior.

**Table 2:** Results of the eye-tracking test.

(A) Results of the eye-tracking test in Conditions A and B, using two-tailed paired-sample *t*-tests														
Eye movement metrics	Humble Administrator’s Garden (Real)					Humble Administrator’s Garden (VR)					*t* (df = 25)	*p*	Effect size (dz)	Power
	Mean	SD	SE	95CI		Mean	SD	SE	95CI					
				LB	UB				LB	UB				
Fixation count	287.481	102.138	19.656	326.008	248.955	347.679	85.063	16.075	379.186	316.171	1.800	0.078	0.355	0.413
Fixation duration	260.684	26.710	5.140	270.759	250.609	206.293	26.718	5.049	216.189	196.396	5.323	0.000**	1.044	0.999
Mean duration(s)	1.022	0.338	0.065	1.150	0.895	0.627	0.180	0.034	0.693	0.560	4.441	0.000**	0.871	0.992
Ratio of fixations	0.455	0.047	0.009	0.473	0.438	0.106	0.038	0.007	0.120	0.092	1.008	0.318	0.199	0.164
Ratio of fixation duration	0.829	0.100	0.019	0.866	0.791	0.687	0.089	0.017	0.720	0.654	4.079	0.000**	0.800	0.979

(B) Results of the eye-tracking test for the uncrowded and crowded groups within Condition A, using the Wilcoxon rank-sum test														
Eye movement metrics	Uncrowded Group						Crowded Group				*Z*	*p*	Effect size (r)	Power
	Mean	SD	SE	95CI		Mean	SD	SE	95CI					
				LB	UB				LB	UB				
Fixation count	278.308	63.040	17.484	312.577	244.039	310.846	130.513	36.198	381.794	239.899	0.832	0.404	0.289	0.227
Fixation duration	271.188	9.417	2.612	276.307	266.069	251.090	22.890	9.380	267.468	230.697	2.116	0.035*	0.574	0.708
Mean duration(s)	1.090	0.263	0.073	1.233	0.947	0.941	0.395	0.110	1.156	0.726	1.085	0.277	0.131	0.085
Ratio of fixations	0.473	0.016	0.004	0.482	0.465	0.435	0.059	0.016	0.467	0.403	2.093	0.036*	0.565	0.775
Ratio of fixation duration	0.857	0.058	0.016	0.888	0.826	0.796	0.125	0.035	0.864	0.728	1.504	0.132	0.310	0.254

*p < 0.05, **p < 0.01.

AOIs analysis using two-tailed paired-sample *t*-tests found that architectural features, plantscape, and waterscape attracted more attention in both real-world and virtual views of the Humble Administrator’s Garden (Figure 4B). Fixations on natural elements like the sky and plant spaces were longer in real-world settings than in VR [Sky: *p* = 0.035, *dz* = 0.437, *power* = 0.824, *t(25)* = 2.228; Plant spaces: *p* = 0.004, *dz* = 0.612, *power* = 0.856, *t(25)* = 3.120]. Fixation count and duration on waterscapes were higher in real-world environments [*p* = 0.047, *dz* = 0.410, *power* = 0.552, *t(25)* = 2.089; *p* = 0.002, *dz* = 0.680, *power* = 0.920, *t*(25) = 3.467]. Although more fixations were directed toward buildings in VR, the duration of these fixations remained longer in the real-world garden [*p* = 0.027, *dz* = 0.461, *power* = 0.614, *t(25)* = 2.350]. Within the real-world condition, Wilcoxon rank-sum tests revealed that the Uncrowded group exhibited longer and more frequent fixations on plant spaces (number: *p* = 0.036, *r* = 0.582, *power* = 0.556, *Z* = 2.100; duration: *p* = 0.023, *r* = 0.630, *power* = 0.622, *Z* = 2.270), suggesting greater engagement with natural elements in quieter settings (Figure 4C). Conversely, the Crowded group showed increased fixations on waterscapes (number: *p* = 0.039, *r* = 0.573, *power* = 0.542, *Z* = 2.066; duration: *p* = 0.041, *r* = 0.566, *power* = 0.533, *Z* = 2.041) and hardscapes (number: *p* = 0.047, *r* = 0.552, *power* = 0.512, *Z* = 1.990; duration: *p* = 0.038, *r* = 0.575, *power* = 0.545, *Z* = 2.074), indicating a shift toward built features under crowding conditions.

**Figure 8:**
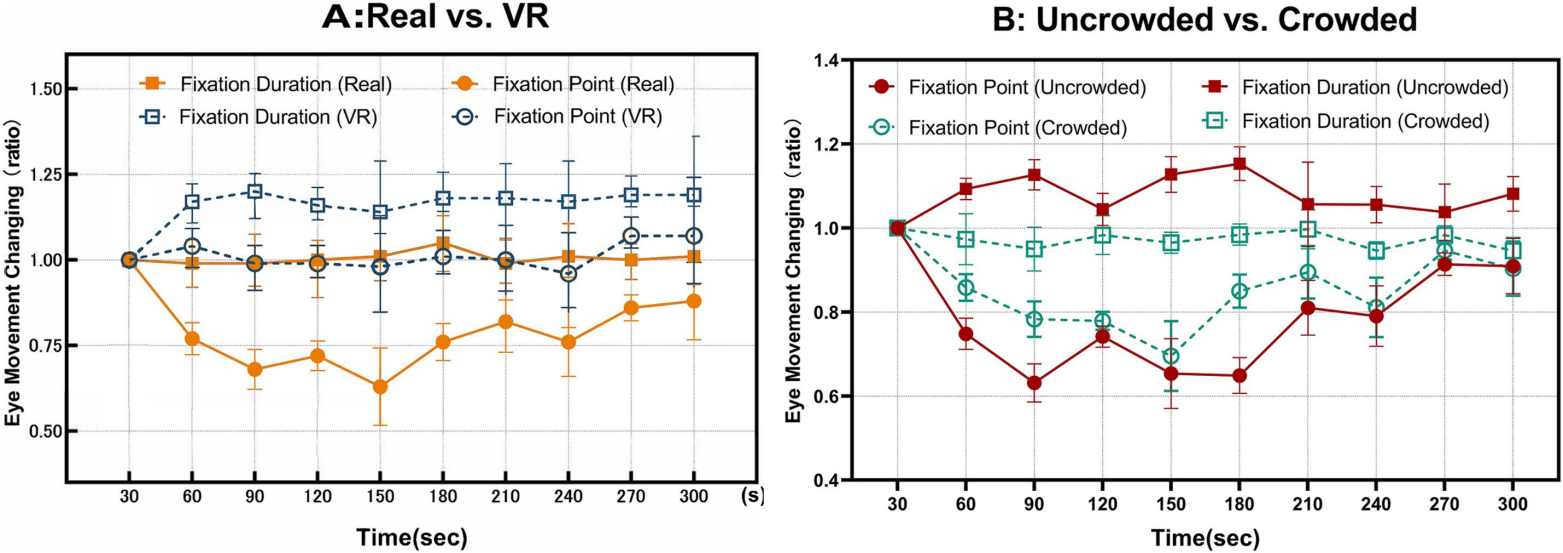
Line graphs of temporal changes in eye-movement metrics. **(A)** Temporal ratio changes for fixation counts and fixation durations in Conditions A and B (*n* = 26). **(B)** Temporal ratio changes for the number of fixations, fixation duration for the Uncrowded and Crowded groups within Condition A (*n* = 13).

Two-way repeated-measures ANOVAs on fixation count and duration revealed a significant main effect of condition [*p* = 0.027, *ηp^2^* = 0.079, *power* = 0.537, *F(1, 25)* = 5.423], with values higher in VR than in the real-world setting (Figure 8A). No main effect of time or time × condition interaction was found (all *ps* > 0.05). Within Condition A, the time × crowding interaction was significant for both metrics, whereas the main effect of crowding was not [count: *p* = 0.024, *ηp^2^* = 0.155, *power* = 0.737, *F(9, 108)* = 2.208; duration: *p* = 0.035, *ηp^2^* = 0.150, *power* = 0.692, *F(9, 108)* = 2.124]. Given the interaction, we examined simple time effects within each crowding level (using a Bonferroni-adjusted within-family approach). In the Uncrowded group, time was significant for both fixation count [*p* = 0.015, ηp^2^ = 0.168, *power* = 0.776, *F(9,108)* = 2.437] and fixation duration [*p* = 0.043, *ηp^2^* = 0.144, *power* = 0.845, *F(9, 108)* = 2.033], indicating a decrease in fixation count alongside an increase in fixation duration over time. In the Crowded group, time was not significant for either metric (Figure 8B). This pattern reflects a shift toward fewer but longer fixations only under uncrowded viewing.

### 4.4 Supplemental questionnaire

The supplemental questionnaire results showed that fewer than 20% of participants were familiar with Chinese classical gardens or VR technology, indicating that most had limited prior exposure (Table 3A).

**Table 3:** Distributions of responses to the supplemental questionnaire.

(A) Familiarity with classical Chinese gardens/VR devices (*n* = 26)											
Question	Condition	Strongly agree		Agree		Neutral		Disagree		Strongly disagree	
		*n*	%	*n*	%	*n*	%	*n*	%	*n*	%
Q1. I am familiar with classical Chinese gardens/VR devices.	Garden	2	7.69%	3	11.53%	20	76.92%	1	3.84%	–	–
	VR device	1	3.84%	3	11.53%	16	61.53%	5	19.23%	1	3.84%

(B) Q2–Q4 by condition: HAG (Real) and HAG (VR), *n* = 26 each; Uncrowded group (UG) and Crowded group (CG) within the real-world condition, *n* = 13 each; HAG = Humble Administrator’s Garden											
Question	Condition	Strongly agree		Agree		Neutral		Disagree		Strongly disagree	
		*n*	%	*n*	%	*n*	%	*n*	%	*n*	%
Q2. I like the view of the garden.	HAG (Real)	18	69.23%	8	30.76%	–	–	–	–	–	–
	HAG (VR)	16	61.54%	7	26.92%	1	3.84%	1	3.84%	1	3.84%
	UG	11	84.62%	2	15.38%	–	–	–	–	–	–
	CG	3	23.08%	10	76.92%	–	–	–	–	–	–
Q3. I want to view this garden again.	HAG (Real)	12	46.15%	10	38.46%	–	–	1	3.84%	–	–
	HAG (VR)	9	34.62%	11	42.31%	1	3.84%	3	11.53%	1	3.84%
	UG	9	69.23%	4	30.77%	–	–	–	–	–	–
	CG	2	15.38%	7	53.85%	–	–	4	30.77%	–	–
Q4. I felt relaxed during this viewing process.	HAG (Real)	18	69.23%	8	30.76%	–	–	–	–	–	–
	HAG (VR)	8	30.76%	11	42.31%	5	19.23%	2	7.69%	–	–
	UG	10	76.92%	3	23.08%	–	–	–	–	–	–
	CG	9	69.23%	–	–	–	–	4	30.77%	–	–

Under Condition A, responses varied by visitor presence. In the Uncrowded group, all liked the garden view (100%) and felt relaxed (100%). In contrast, in the Crowded group, only 3 participants (23%) reported liking the view, and 9 participants (69%) reported feeling relaxed, while 4 participants (31%) did not (Table 3B). Fisher’s exact tests confirmed significant differences between Uncrowded and Crowded groups for liking the garden view (Q2, *p* = 0.0048) and willingness to view the garden again (Q3, *p* = 0.015), with a non-significant trend observed for relaxation (Q4, *p* = 0.096). These findings suggest crowd-related disturbances reduce positive responses.

Open-ended responses revealed differences across settings. In the garden, many described it as “like a traditional landscape painting,” “poetic,” or “a historical scene,” and reported it encouraged reflection and associative thinking. In the VR condition, experiences were often described as “novel” but later “static,” “emotionally flat,” or “uneasy.” One participant noted, *“The visuals were fine, but after a while it felt empty—like something was missing.”* Feedback also differed between Uncrowded and Crowded groups. The uncrowded participants provided more thoughtful, culturally meaningful comments, while the crowded group offered simpler remarks about distractions, such as “too noisy,” “regrettable,” and “uneasy.” One participant explained, *“At first, the view was awe-inspiring to me, but later, the crowds and conversations around me made me feel uncomfortable and guilty about impeding other visitors’ passage and sightlines.”* These responses suggest crowd disturbances influenced both relaxation and cultural or emotional engagement.

**Figure 9:**
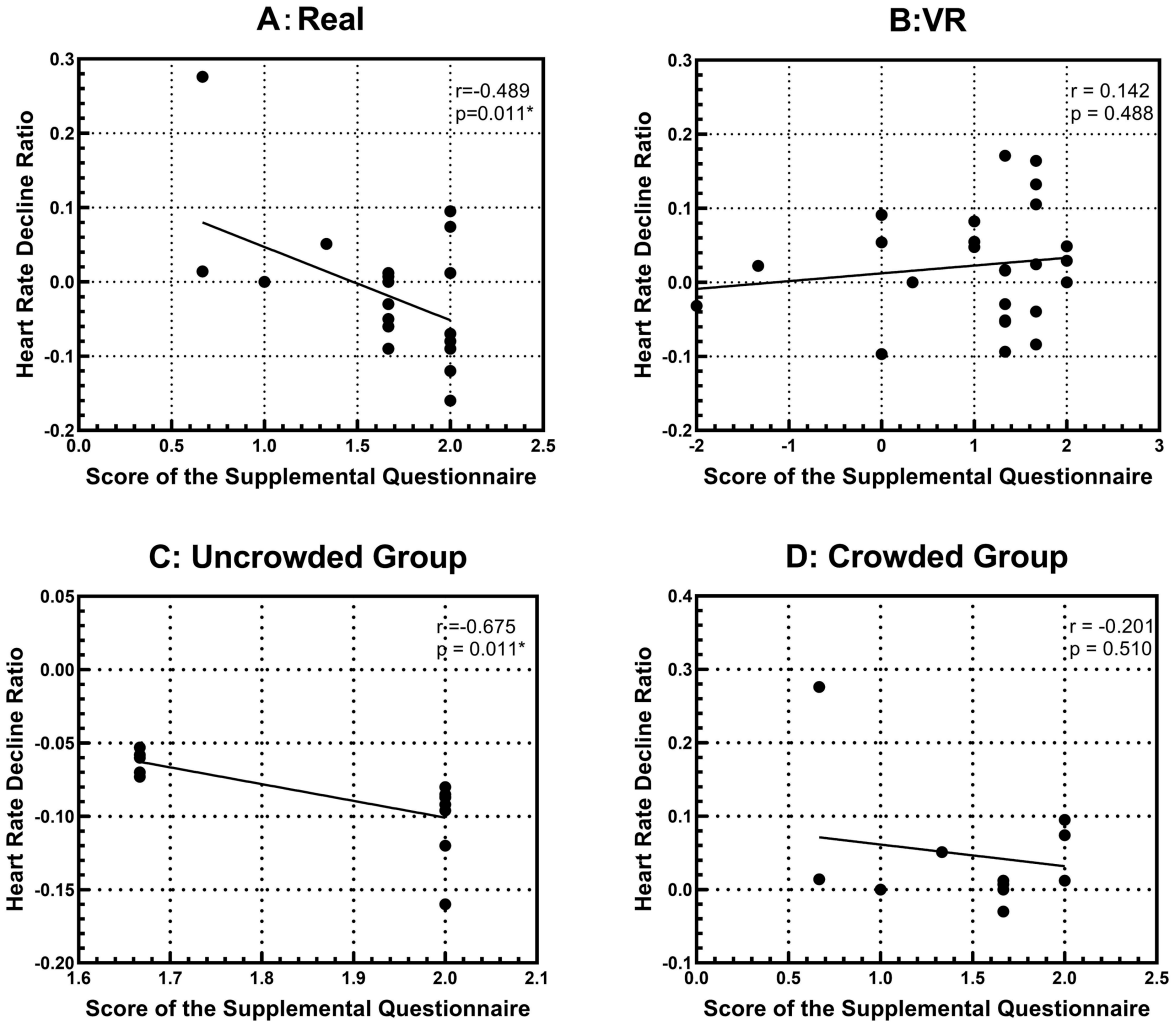
Correlation graph of heart rate and questionnaire. Baseline = 1.0. **(A)** Correlation between heart rate decline ratio and questionnaire responses in Condition A. **(B)** Correlation between heart rate decline ratio and questionnaire responses in Condition B. **(C)** Correlation between heart rate decline ratio and questionnaire responses for the Uncrowded group in Condition A. **(D)** Correlation between heart rate decline ratio and questionnaire responses for the Crowded group in Condition A.

Further analysis explored the link between heart rate reduction during a 5-min viewing and questionnaire responses. Figure 9 shows the association between subjective appraisals and physiological restoration. A significant negative correlation was found in Condition A [*p =* 0.011, *r* = −0.489, *power* = 0.781, *t(24) = 2.747*], indicating that more positive ratings were linked to greater heart rate decline (Figure 9A). This association was particularly evident in the Uncrowded group [*p =* 0.012, *r* = −0.669, *power* = 0.893, *t(11) = 2.985*], with a clear connection between impressions and responses (Figure 9C). No significant correlations appeared in VR or the Crowded group (Figures 9B,D), where impressions did not match heart rate changes. Overall, the link between subjective and physiological restoration was strongest in a quiet, uncrowded environment, but weakened with VR or crowding.

## 5 Discussion

### 5.1 Restorative differences between real-world and virtual garden experiences

Consistent with theories of multisensory integration and presence (Calvert et al., 2004; Spence, 2020a,b; Slater and Wilbur, 1997), the results indicated that real-world exposure produced greater attentional engagement, emotional balance, and physiological relaxation compared with the static visual-only VR condition. In the real-world garden, coherent visual, auditory, olfactory, and tactile cues supported sustained attention and soft fascination, whereas the VR scene lacked cross-modal coherence.

Eye-tracking data further substantiated the restorative distinction between real-world and virtual experiences. In the real-world garden, participants’ gaze reflected smoother and more sustained engagement, characterized by stable attention to meaningful landscape elements such as vegetation, water, and sky. This suggests that natural, multisensory environments facilitate effortless attention and deeper perceptual engagement, aligning with the concept of “soft fascination” in restorative settings (Kaplan and Kaplan, 1989). In contrast, gaze behavior in the VR condition appeared more fragmented and spatially confined, suggesting reduced perceptual immersion and attentional continuity within the static, visual-only scene (Rayner, 2009). These attentional dynamics were not limited to visual metrics but extended to physiological and affective responses. Physiological and psychological responses converged with these visual patterns. The real-world environment facilitated a more pronounced relaxation process, reflected in a gradual decline observed in the uncrowded real-world garden conditions and in benefits to mood, while the VR condition produced only partial benefits (Ulrich et al., 1991). Taken together, the findings suggest that coherent multisensory stimulation enhances attentional stability, emotional balance, and autonomic recovery.

Participants’ spontaneous descriptions of the real-world garden as “poetic” and “painterly” appear consistent with the design principle of *Yijing* (意境)—a poetic and immersive atmosphere created through layered sensory experiences. Such coherence between visual composition and other sensory cues may facilitate reflection and emotional renewal (Wang, 2015; Wang, 2023; Zheng et al., 2024). Notably, restorative responses occurred even among participants unfamiliar with classical symbolism, indicating that multisensory coherence itself, rather than explicit cultural knowledge, could be a major contributor to the restorative experience. Nevertheless, cultural framing may amplify these sensory effects, which warrants further empirical investigation.

### 5.2 Impact of crowding on real-world garden restoration

Although the influx of tourists caused disturbances, it also provided insights. The data showed that viewing the classical garden in a real-world setting led to stronger restorative effects. Comparing Uncrowded and Crowded groups highlighted differences: the Uncrowded group reported lower Depression and Fatigue, consistent with expectations for restorative environments (Lestari and Favurita, 2024). In the participants’ comments, those in the Uncrowded group offered more thoughtful and associative responses, while those in the Crowded group gave simpler comments with more negative phrases. This context is important for understanding the physiological and behavioral outcomes.

Physiological and behavioral data supported these differences. Eye-tracking showed that participants in the Uncrowded group had broader scan paths and longer fixations, indicating deeper visual engagement (Leszczynski et al., 2020). Fixation patterns evolved, with fewer but longer fixations, suggesting a gradual shift toward more sustained and effortless engagement with the scene (Antes, 1974; Henderson and Hollingworth, 1999). In contrast, the crowded group had shorter fixations and focused mainly on hardscape features, likely due to increased cognitive effort managing auditory and visual distractions in the crowded environment (Nishino et al., 2013; Zvyagintsev et al., 2013; Andersson et al., 2018; Eghdam et al., 2020). These findings are consistent with research on sustained attention and beneficial mood modulation, which emphasizes that stable attentional engagement supports both affective balance and cognitive integration (Posner and Rothbart, 2007; Gross, 2015). Heart rate data indicated the Uncrowded group’s arousal declined throughout, while the crowded group’s arousal decreased initially then rebounded, possibly due to ongoing crowd noise and movement (Singer et al., 2016; Yang et al., 2019; Peters et al., 2021).

These findings demonstrate that sensory and aesthetic engagement in classical Chinese gardens depends on visual quality and multisensory harmony. In uncrowded conditions, participants were able to sustain attention longer, engage in reflective thinking, and experience more complete psychological and physiological recovery. Crowding disrupted this process, with heavy crowds and intrusive visitor-generated sounds breaking sensory harmony and reducing the emotional depth that the garden was designed to evoke.

### 5.3 Limitations and future research directions

The study was conducted in the Humble Administrator’s Garden, a highly representative and iconic site. However, using only this location limits capturing the diversity of classical garden design features. The small sample size, limited by site access and coordination in a heritage garden, decreased statistical power and generalizability. Participants were mainly landscape university students, limiting diversity and external validity. The within-subjects design may have caused sensitization or order effects affecting responses. Results suggest links between restorative outcomes and cognitive processes like attention or associative thinking, but these were not directly measured. The VR condition, based on a static 360° image, lacked natural motion, reducing realism compared to the real-world garden. Additionally, presence influences engagement, enjoyment, and restorative effects, so not assessing it may limit VR result interpretation.

Future research should first recruit larger, more diverse samples and adopt designs that better control order effects to increase statistical power and internal validity. Next, we suggest incorporating direct cognitive measures and testing across multiple gardens with varied profiles can improve construct coverage and generalizability. In parallel, we recommend using advanced VR that manipulates visual motion, spatial audio, olfactory input, airflow, and navigation can narrow the gap with real-world settings and identify the contribution of each modality. Simultaneously, include standardized presence scales like the Igroup Presence Questionnaire can measure immersion and clarify how presence influences responses. Finally, longitudinal VR studies with input from environmental psychology, landscape architecture, and immersive tech can improve strategies for physical and virtual restorative spaces. These steps will promote culturally authentic, multisensory uses in therapy, education, and conservation.

### 5.4 Theoretical and practical contributions

As a pilot study testing our core hypothesis, the results consistently showed increased attentional engagement, emotional regulation, and physiological relaxation in the real-world, multisensory garden compared to the static VR setting. This highlights how non-visual cues enhance coherence, attention, and emotional response.

Theoretically, these findings support Attention Restoration Theory and Stress Recovery Theory by showing that sustained visual engagement mediates the link between multisensory exposure and restorative outcomes. These findings also align with multisensory integration and presence theories, which posit that coherent, richly cued environments elicit stronger attentional and affective responses than static, single-modality scenes.

Methodologically, integrating eye-tracking, heart rate, and mood measures within a culturally specific landscape demonstrates the feasibility of a multi-modal approach to quantifying restoration. The static 360° VR setting, while limited, offers a controlled reference for future progression toward more immersive designs.

Practically, although visual-only VR lacks sound, scent, and natural motion, its partial mood benefits suggest potential value in mental-health interventions and educational contexts where direct access to natural environments is constrained. Additionally, exploratory results indicate that visitor density and noise may reduce restoration, implying that managing crowding and acoustic conditions could help maintain the restorative value of heritage sites.

In summary, this study bridges landscape architecture, environmental psychology, and heritage management. Multisensory richness emerges as a key driver of restorative experience. At the same time, the framework presented here provides a conceptual and methodological basis for cross-site investigations and for advancing immersive, evidence-based VR applications that support psychological restoration.

## Data Availability

The raw data supporting the conclusions of this article will be made available by the authors, without undue reservation.
