# Supplementary material for: Restorative benefits of multisensory experiences in a classical Chinese garden compared to visual experiences only
Source: Front Psychol. 2025 Nov 28;16:1663101. doi: 10.3389/fpsyg.2025.1663101 (PMC12698485; doi:10.3389/fpsyg.2025.1663101)
Supplement: Supplementary file 1 [file Table_1.DOCX]

**Appendix A**


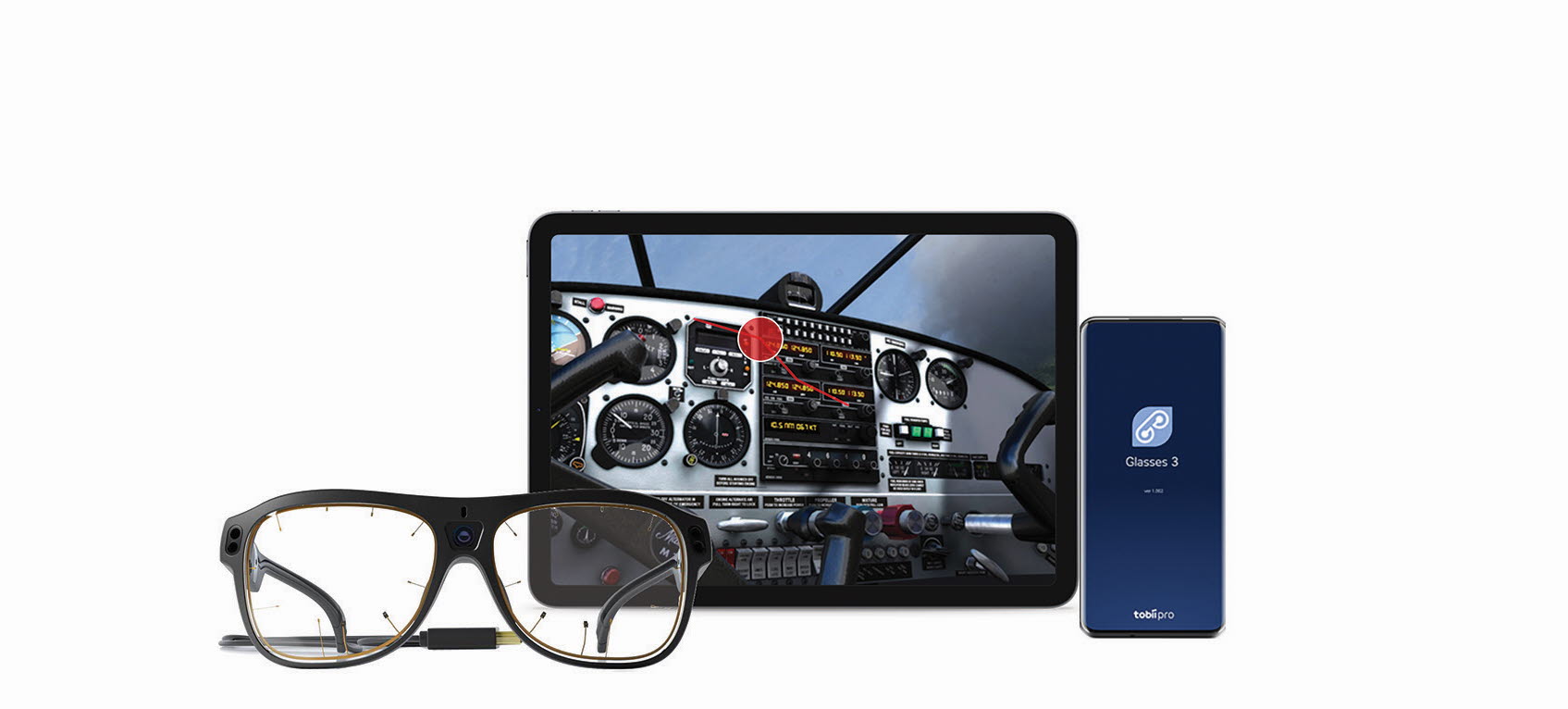


**Plate 1** Eye tracking device: Tobii Pro Glasses 3, employed in condition A experiments to investigate gaze behavior and visual attention in real-world settings. <https://www.tobii.com/ja/products/eye-trackers/wearables/tobii-pro-glasses-3>


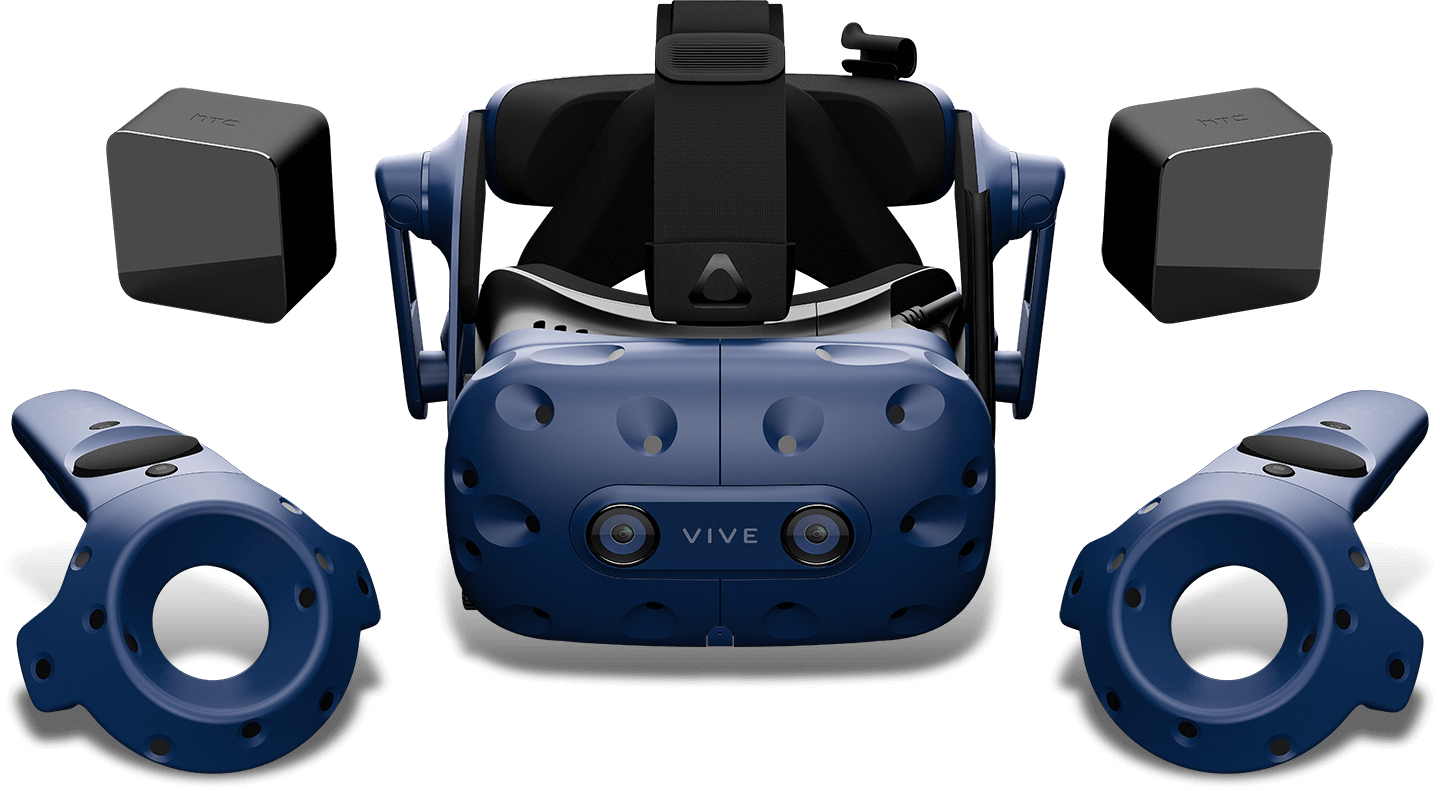


**Plate 2** Eye tracking device: HTC VIVE Pro, utilized in condition B experiments to assess visual attention and gaze behavior in VR settings.

<https://www.tobii.com/ja/products/eye-trackers/wearables/tobii-pro-glasses-3>

**
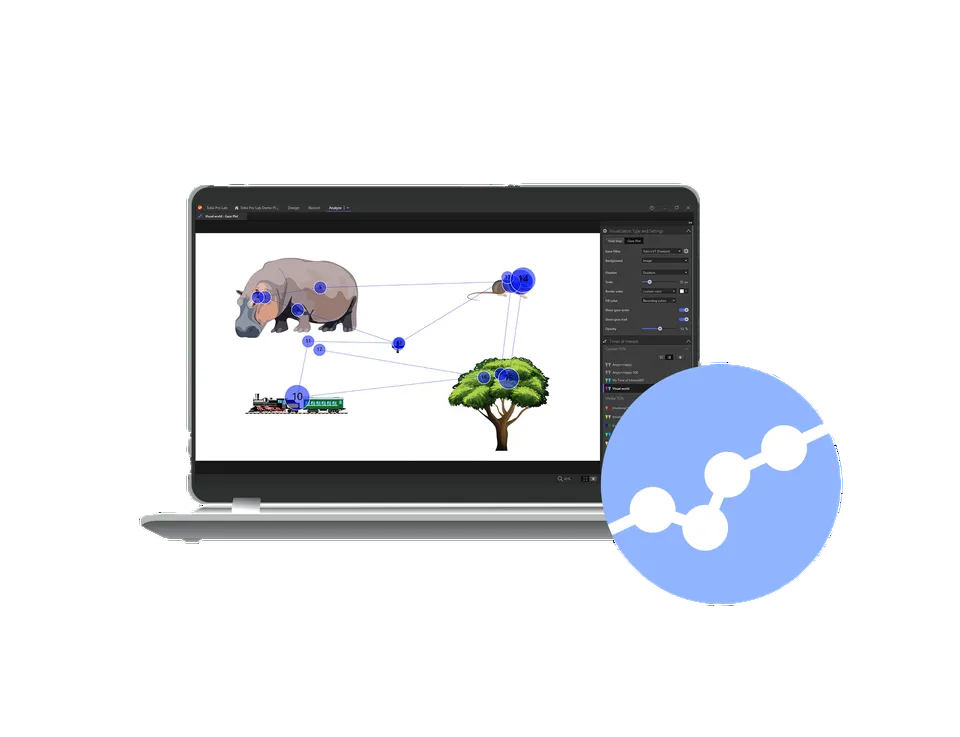
Plate 3** Eye movement data analytics software. Tobii Pro Lab is an analysis software that supports data from Tobii eye-tracking devices, including screen-based, wearable, and VR systems, enabling consistent processing and analysis of gaze metrics.

<https://www.tobii.com/products/software/behavior-research-software/tobii-pro-lab>
