# Supplementary material for: Restorative benefits of multisensory experiences in a classical Chinese garden compared to visual experiences only
Source: Front Psychol. 2025 Nov 28;16:1663101. doi: 10.3389/fpsyg.2025.1663101 (PMC12698485; doi:10.3389/fpsyg.2025.1663101)
Supplement: Supplementary file 2 [file Table_2.DOCX]

**Appendix B**

**Profile of Mood States (POMS) Questionnaire**

Date:

Age:

Gender:

Participant number:

|  | Not At All | A Little | Moderately | Quite a lot | Extremely |
| --- | --- | --- | --- | --- | --- |
| 1.Tense | 0 | 1 | 2 | 3 | 4 |
| 2.Angry | 0 | 1 | 2 | 3 | 4 |
| 3.Worn Out | 0 | 1 | 2 | 3 | 4 |
| 4.Lively | 0 | 1 | 2 | 3 | 4 |
| 5.Confused | 0 | 1 | 2 | 3 | 4 |
| 6. Unable to concentrate | 0 | 1 | 2 | 3 | 4 |
| 7. Sad | 0 | 1 | 2 | 3 | 4 |
| 8. Active | 0 | 1 | 2 | 3 | 4 |
| 9. Grouchy | 0 | 1 | 2 | 3 | 4 |
| 10. Energetic | 0 | 1 | 2 | 3 | 4 |
| 11. Ashamed | 0 | 1 | 2 | 3 | 4 |
| 12. Uneasy | 0 | 1 | 2 | 3 | 4 |
| 13. Fatigued | 0 | 1 | 2 | 3 | 4 |
| 14. Sorry for things done | 0 | 1 | 2 | 3 | 4 |
| 15.Discouraged | 0 | 1 | 2 | 3 | 4 |
| 16. Nervous | 0 | 1 | 2 | 3 | 4 |
| 17. Lonely | 0 | 1 | 2 | 3 | 4 |
| 18. Uncertain about things | 0 | 1 | 2 | 3 | 4 |
| 19. Exhausted | 0 | 1 | 2 | 3 | 4 |
| 20.Anxious | 0 | 1 | 2 | 3 | 4 |
| 21.Blue | 0 | 1 | 2 | 3 | 4 |
| 22.Peeved | 0 | 1 | 2 | 3 | 4 |
| 23.Restless | 0 | 1 | 2 | 3 | 4 |
| 24.Bushed | 0 | 1 | 2 | 3 | 4 |
| 25.Resentful | 0 | 1 | 2 | 3 | 4 |
| 26.Confident | 0 | 1 | 2 | 3 | 4 |
| 27. Full of Pep | 0 | 1 | 2 | 3 | 4 |
| 28. Furious | 0 | 1 | 2 | 3 | 4 |
| 29. Forgetful | 0 | 1 | 2 | 3 | 4 |
| 30. Vigorous | 0 | 1 | 2 | 3 | 4 |

**The Supplemental Questionnaire**

Date:

Age:

Gender:

Participant number:

1. **I am familiar with classical Chinese gardens /VR devices.**

- Strongly agree
- Agree
- Neither agree nor disagree
- Disagree
- Strongly disagree

1. **I like the view of the garden.**

- Strongly agree
- Agree
- Neither agree nor disagree
- Disagree
- Strongly disagree

1. **I want to view this garden again.**

- Strongly agree
- Agree
- Neither agree nor disagree
- Disagree
- Strongly disagree

1. **I felt relaxed during the viewing process.**

- Strongly agree
- Agree
- Neither agree nor disagree
- Disagree
- Strongly disagree

1. **Please provide any additional thoughts or comments in the space below.**

＿＿＿＿＿＿＿＿＿＿＿＿

＿＿＿＿＿＿＿＿＿＿＿＿

＿＿＿＿＿＿＿＿＿＿＿＿

＿＿＿＿＿＿＿＿＿＿＿＿

＿＿＿＿＿＿＿＿＿＿＿＿

＿＿＿＿＿＿＿＿＿＿＿＿

＿＿＿＿＿＿＿＿＿＿＿＿
