# Supplementary material for: Restorative benefits of multisensory experiences in a classical Chinese garden compared to visual experiences only
Source: Front Psychol. 2025 Nov 28;16:1663101. doi: 10.3389/fpsyg.2025.1663101 (PMC12698485; doi:10.3389/fpsyg.2025.1663101)
Supplement: Supplementary file 3 [file Table_3.DOCX]

**Tables**

| **1-1** |  |  |  |  |  |  |  |  |
| --- | --- | --- | --- | --- | --- | --- | --- | --- |
|  | **MEAN ± SD  (Real)** | **MEAN ± SD  (VR)** | **Mean Diff.  (Real – VR)** | **SE Diff.  (Real – VR)** | **t  (df = 25)** | ***p*** | **Effect size (*dz*)** | ***power*** |
| **T-A** | −5.947 ± 10.63 | −4.645 ± 6.63 | −1.302 | 0.852 | −1.525 | 0.14016 | 0.299 | 0.311 |
| **D** | −4.191 ± 13.88 | −3.356 ± 10.30 | −0.835 | 1.079 | −0.769 | 0.449 | 0.151 | 0.114 |
| **A-H** | −6.615 ± 9.47 | −1.114 ± 8.00 | −5.509 | 2.143 | −2.571 | 0.016* | 0.504 | 0.695 |
| **V** | 0.948± 9.61 | 1.654 ± 7.93 | −0.796 | 0.804 | −0.871 | 0.392 | 0.171 | 0.133 |
| **F** | −8.840 ± 11.27 | −8.959 ± 10.06 | 0.119 | 0.265 | 0.452 | 0.655 | 0.089 | 0.072 |
| **C** | −2.434 ± 7.15 | −1.236 ± 6.61 | −1.192 | 0.434 | −2.742 | 0.011* | 0.538 | 0.751 |
|  |  |  |  |  |  |  |  |  |
| **1-2** |  |  |  |  |  |  |  |  |
|  | **MEAN ± SD  (Uncrowded)** | **MEAN ± SD  (Crowded)** | **Mean Diff.  (Uncrowded – Crowded)** | ***U*** | ***Z*** | ***p*** | **Effect size (*r*)** | ***power*** |
| **T-A** | −1.026 ± 9.328 | −1.940 ± 9.428 | 0.914 | 69.500 | 0.776 | 0.439 | 0.152 | 0.061 |
| **D** | −2.530 ± 6.230 | −0.121 ± 9.054 | −2.409 | 81.000 | 0.176 | 0.859 | 0.035 | 0.107 |
| **A-H** | −0.294 ± 4.024 | −2.778 ± 5.932 | 2.484 | 26.500 | 2.967 | 0.003** | 0.582 | 0.819 |
| **V** | 1.887 ± 9.748 | 1.565 ± 16.002 | 0.322 | 82.000 | 0.126 | 0.899 | 0.025 | 0.053 |
| **F** | −4.222 ± 7.010 | 1.571 ± 7.236 | −5.793 | 46.500 | 1.943 | 0.052 | 0.381 | 0.344 |
| **C** | −0.542 ±11.399 | 0.004 ± 10.942 | −0.546 | 83.500 | 0.063 | 0.947 | 0.012 | 0.052 |

**Table 1.** **Results of Profile of Mood States Questionnaire (post–pre).** Table 1-1. Results of Profile of Mood States Questionnaire in the experiments of Condition A and B, using two-tailed paired-sample t-tests. Table 1-2. Results of Profile of Mood States Questionnaire for the uncrowded and crowded group within Condition A, using the Wilcoxon rank-sum test**. T-A** = Tension-anxiety, **D** = Depression-dejection, **A-H** = Anger-hostility, **V** = Vigor-activity, **F** =Fatigue-inertia, and **C** = Confusion-bewilderment (* p < 0.05, ** p < 0.01).

| **2-1** |  |  |  |  |  |  |  |  |  |  |  |  | |  | |  | |  | |  |  |  |
| --- | --- | --- | --- | --- | --- | --- | --- | --- | --- | --- | --- | --- | --- | --- | --- | --- | --- | --- | --- | --- | --- | --- |
|  | **Humble Administrator’s Garden（Real）** | | | | |  | **Humble Administrator’s Garden (VR)** | | | | | |  | |  | |  | |  |  | |  |
|  | **MEAN** | **SD** | **SE** | **95CI** | |  | **MEAN** | **SD** | **SE** | **95CI** | |  | | **t (26)** | | ***p*** | | ***Effect size (d)*** | | ***Power*** | | |
|  |  |  |  | **LB** | **UB** |  |  |  |  | **LB** | **UB** |  | |  |  |  |  |  |  |  |  |  |
| The number of fixations | 287.481 | 102.138 | 19.656 | 326.008 | 248.955 |  | 347.679 | 85.063 | 16.075 | 379.186 | 316.171 |  | | 1.800 | | 0.078 | | 0.355 | | 0.413 |  |  |
| The fixation duration | 260.684 | 26.710 | 5.140 | 270.759 | 250.609 |  | 206.293 | 26.718 | 5.049 | 216.189 | 196.396 |  | | 5.323 | | 0.000** | | 1.044 | | 0.999 |  |  |
| Mean duration(s) | 1.022 | 0.338 | 0.065 | 1.150 | 0.895 |  | 0.627 | 0.180 | 0.034 | 0.693 | 0.560 |  | | 4.441 | | 0.000** | | 0.871 | | 0.992 |  |  |
| Ratio of fixations | 0.455 | 0.047 | 0.009 | 0.473 | 0.438 |  | 0.106 | 0.038 | 0.007 | 0.120 | 0.092 |  | | 1.008 | | 0.318 | | 0.199 | | 0.164 |  |  |
| Ratio of fixation duration | 0.829 | 0.100 | 0.019 | 0.866 | 0.791 |  | 0.687 | 0.089 | 0.017 | 0.720 | 0.654 |  | | 4.079 | | 0.000** | | 0.800 | | 0.979 |  |  |

| **2-2** |  |  |  |  |  |  |  |  |  |  |  |  |  |  |  |  |
| --- | --- | --- | --- | --- | --- | --- | --- | --- | --- | --- | --- | --- | --- | --- | --- | --- |
|  |  | Uncrowded Group | | |  |  |  | Crowded Group | | |  |  |  |  |  |  |
|  | **MEAN** | **SD** | **SE** | **95CI** | |  | **MEAN** | **SD** | **SE** | **95CI** | |  | ***Z*** | ***p*** | ***Effect size (r)*** | ***Power*** |
|  |  |  |  | **LB** | **UB** |  |  |  |  | **LB** | **UB** |  |  |  |  |  |
| The number of fixations | 278.308 | 63.040 | 17.484 | 312.577 | 244.039 |  | 310.846 | 130.513 | 36.198 | 381.794 | 239.899 |  | 0.832 | 0.404 | 0.289 | 0.227 |
| The fixation duration | 271.188 | 9.417 | 2.612 | 276.307 | 266.069 |  | 251.090 | 22.890 | 9.380 | 267.468 | 230.697 |  | 2.116 | 0.035* | 0.574 | 0.708 |
| Mean duration(s) | 1.090 | 0.263 | 0.073 | 1.233 | 0.947 |  | 0.941 | 0.395 | 0.110 | 1.156 | 0.726 |  | 1.085 | 0.277 | 0.131 | 0.085 |
| Ratio of fixations | 0.473 | 0.016 | 0.004 | 0.482 | 0.465 |  | 0.435 | 0.059 | 0.016 | 0.467 | 0.403 |  | 2.093 | 0.036* | 0.565 | 0.775 |
| Ratio of fixation duration | 0.857 | 0.058 | 0.016 | 0.888 | 0.826 |  | 0.796 | 0.125 | 0.035 | 0.864 | 0.728 |  | 1.504 | 0.132 | 0.310 | 0.254 |

**Table 2. Results of the Eye-Tracking Test.** Table 2-1. Results of the Eye-Tracking Test in Conditions A and B, using two-tailed paired-sample t-tests. Table 2-2. Results of the Eye-Tracking Test for the uncrowded and crowded groups within Condition A, using the Wilcoxon rank-sum test (* p < 0.05, ** p < 0.01.

| **3-1** |  |  | |  | |  | |  | |  | |
| --- | --- | --- | --- | --- | --- | --- | --- | --- | --- | --- | --- |
| **Question** | **Condition** | **Strongly agree** | | **Agree** | | **Neutral** | | **Disagree** | | **Strongly disagree** | |
|  |  | **n** | **%** | **n** | **%** | **n** | **%** | **n** | **%** | **n** | **%** |
| **Q1. I am familiar with classical Chinese gardens / VR devices.** | Garden | 2 | 7.69% | 3 | 11.53% | 20 | 76.92% | 1 | 3.84% | – | – |
|  | VR device | 1 | 3.84% | 3 | 11.53% | 16 | 61.53% | 5 | 19.23% | 1 | 3.84% |
|  |  |  |  |  |  |  |  |  |  |  |  |
| **3-2** |  |  |  |  |  |  |  |  |  |  |  |
| **Question** | **Condition** | **Strongly agree** | | **Agree** | | **Neutral** | | **Disagree** | | **Strongly disagree** | |
|  |  | **n** | **%** | **n** | **%** | **n** | **%** | **n** | **%** | **n** | **%** |
| **Q2. I like the view of the garden.** | HAG (Real) | 18 | 69.23% | 8 | 30.76% | – | – | – | – | – | – |
|  | HAG (VR) | 16 | 61.54% | 7 | 26.92% | 1 | 3.84% | 1 | 3.84% | 1 | 3.84% |
|  | UG | 11 | 84.62% | 2 | 15.38% | – | – | – | – | – | – |
|  | CG | 3 | 23.08% | 10 | 76.92% | – | – | – | – | – | – |
| **Q3. I want to view this garden again.** | HAG (Real) | 12 | 46.15% | 10 | 38.46% | – | – | 1 | 3.84% | – | – |
|  | HAG (VR) | 9 | 34.62% | 11 | 42.31% | 1 | 3.84% | 3 | 11.53% | 1 | 3.84% |
|  | UG | 9 | 69.23% | 4 | 30.77% | – | – | – | – | – | – |
|  | CG | 2 | 15.38% | 7 | 53.85% | – | – | 4 | 30.77% | – | – |
| **Q4. I felt relaxed during this viewing process.** | HAG (Real) | 18 | 69.23% | 8 | 30.76% | – | – | – | – | – | – |
|  | HAG (VR) | 8 | 30.76% | 11 | 42.31% | 5 | 19.23% | 2 | 7.69% | – | – |
|  | UG | 10 | 76.92% | 3 | 23.08% | – | – | – | – | – | – |
|  | CG | 9 | 69.23% | – | – | – | – | 4 | 30.77% | – | – |

**Table 3. Distributions of responses to the supplemental questionnaire.** Table 3-1. familiarity with classical Chinese gardens / VR devices (n = 26). Table 3-2. Q2–Q4 by condition: HAG (Real) and HAG (VR), n = 26 each; Uncrowded group (UG) and Crowded group (CG) within the real-world condition, n = 13 each, HAG = Humble Administrator’s Garden.
